# A Low-Cost Insertion Trainer for Resuscitative Endovascular Balloon Occlusion of the Aorta (REBOA)

**DOI:** 10.7759/cureus.9729

**Published:** 2020-08-13

**Authors:** Ryan Walsh, Charles Lei, Kenneth H Palm, Ryan J Van Nostrand, Zachary Sletten

**Affiliations:** 1 Department of Emergency Medicine, Vanderbilt University Medical Center, Nashville, USA; 2 Department of Emergency Medicine, San Antonio Military Medical Center, San Antonio, USA

**Keywords:** reboa, simulation, trainer, simvention, trauma, austere, inexpensive

## Abstract

Resuscitative endovascular balloon occlusion of the aorta (REBOA) is an emergency procedure designed to treat non-compressible torso hemorrhage. Because this is a high-acuity low-occurrence event, it is difficult to train providers on the procedure and difficult for trained providers to stay proficient. Our primary objective was to develop a low-cost, high-fidelity teaching model to increase emergency medicine (EM) resident knowledge, confidence, and proficiency in performing REBOA. We utilized readily available materials to allow for ease of replication and cost-effectiveness. The aorta was simulated by a bicycle tire inner tube, and the femoral artery was simulated by natural rubber tubing. Once connected, these simulated vascular structures were threaded through a plastic torso mold and filled with simulated blood. Participants then performed the REBOA procedure with very little time required for reset between participants. After completing the training using our model, participants completed a survey rating aspects of the session on a five-point Likert scale. Participants included 21 EM residents from all levels of training. Participants rated the fidelity of the REBOA insertion trainer very highly (mean = 4.05, SD 0.67) and felt that the training was overall very useful (mean = 4.29, SD 0.56). Comments regarding the model were universally positive. We present a novel low-cost REBOA task trainer that is easy to build, reusable, and portable, and can be utilized either in a hospital or austere training environment.

## Introduction

Non-compressible torso hemorrhage (NCTH) is a life-threatening emergency that carries a mortality rate of 44.6% [1]. It is a significant cause of morbidity and mortality in both the civilian and military settings. One intervention that has been developed to help treat patients with NCTH is resuscitative endovascular balloon occlusion of the aorta (REBOA) [2-5]. During this procedure, a balloon catheter is inserted into the femoral artery and, once inflated, stops otherwise uncontrollable bleeding until the patient can be taken to the operating room or other definitive treatment can be provided. In the United States, REBOA is typically performed in trauma centers by trauma surgeons or specially trained emergency physicians [6-8]. The placement of an ER-REBOA^TM^ (Prytime Medical, Boerne, TX) catheter is a high-acuity low-occurrence (HALO) event, making it difficult to train providers on the procedure and difficult for trained providers to stay proficient [4]. While commercial task trainers are available, educators are limited by the expense as well as availability of these trainers [9,10]. We have developed, to our knowledge, the first non-commercial, portable and affordable insertion trainer for REBOA. 

## Technical report

Our primary objective was to develop a low-cost, high-fidelity teaching model to increase resident knowledge, confidence, and proficiency in performing REBOA. The project took place at our monthly emergency medicine (EM) residency simulation conference. Our residency is based at an urban level 1 trauma center with over 60,000 patient encounters per year. In 2018, our center took care of 7,800 trauma patients and had 28 REBOA insertions.

**Figure 1:**
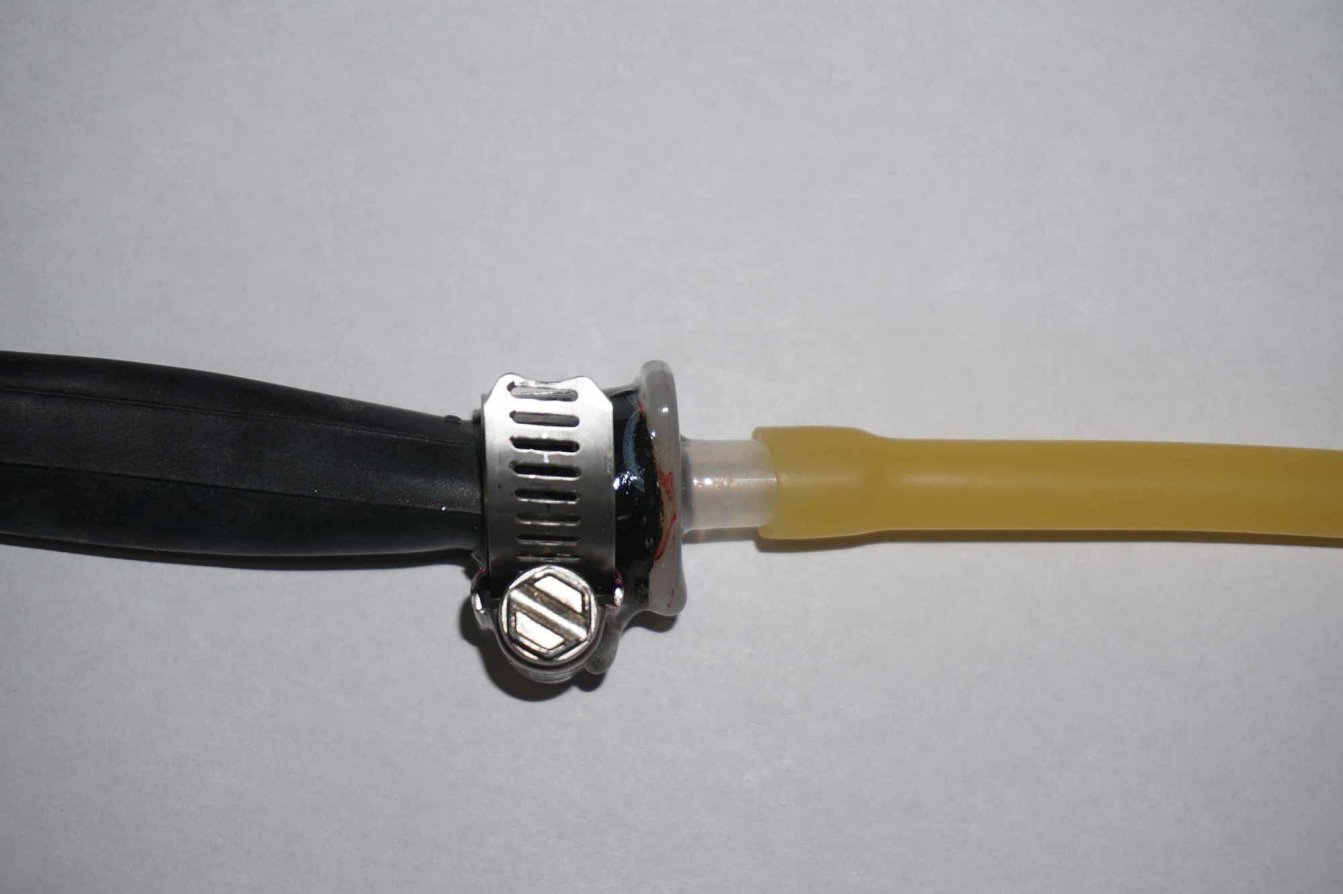
Caudad end of REBOA model. Natural rubber tubing (right, used to simulate the femoral artery) and a road bike tire inner tube (left, used to simulate the aorta) are connected via a 7.5 endotracheal tube connector and a hose clamp. REBOA, resuscitative endovascular balloon occlusion of the aorta

**Figure 2:**
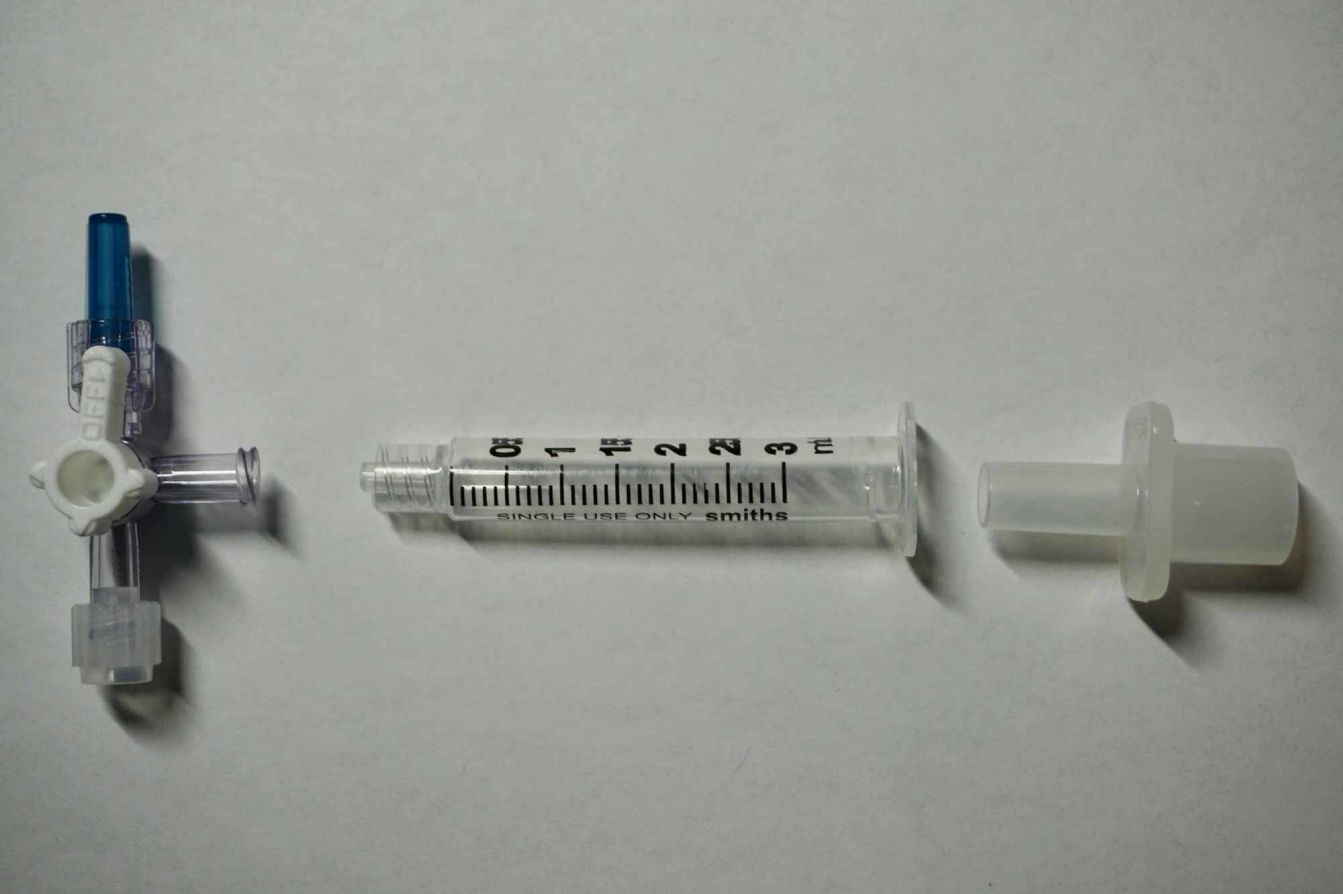
Cephalad end of REBOA model. The bike tire inner tube is connected via a 7.5 endotracheal tube connector (right) to a 3 ml Luer Lock syringe (middle) and a three-way stopcock (left). REBOA, resuscitative endovascular balloon occlusion of the aorta

Our model was created with readily available materials in an effort to allow for ease of replication and cost-effectiveness. The aorta was simulated by a 30-inch length of 25-mm road bicycle tire inner tube and the femoral artery with a 15-inch length of natural rubber tubing with a 3/8-inch outer diameter and 1/4-inch inner diameter. These two sections were connected via a 7.5 endotracheal tube (ETT) connector with a hose clamp and sealant to maintain a sealed system at the caudad end of the model (Figure 1). Similarly, the cephalad end of the inner tube was connected via a 7.5 ETT connector to a 3 ml Luer Lock syringe and three-way stopcock (Figure 2); the ETT connector was sealed and hose clamped in a similar fashion. The overall system was closed with a simple knot in the natural rubber tubing and subsequently filled with red colored liquid through the Luer Lock syringe.

**Figure 3:**
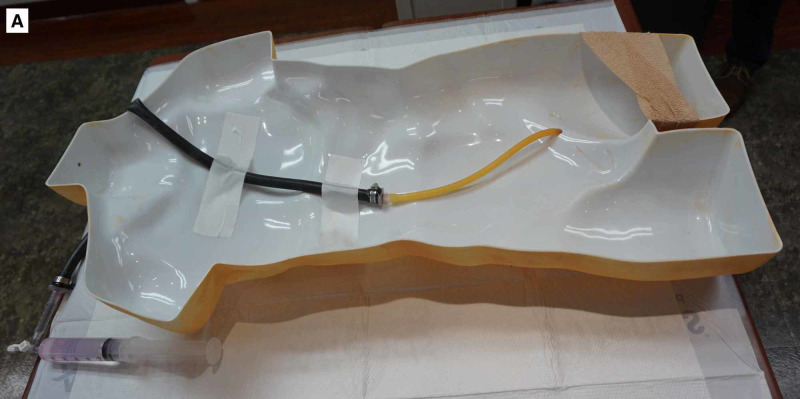
Completed REBOA insertion trainer. The REBOA model is attached to the underside of a human torso mold, with the caudad end passing through the torso at the inguinal region. REBOA, resuscitative endovascular balloon occlusion of the aorta

**Figure 4:**
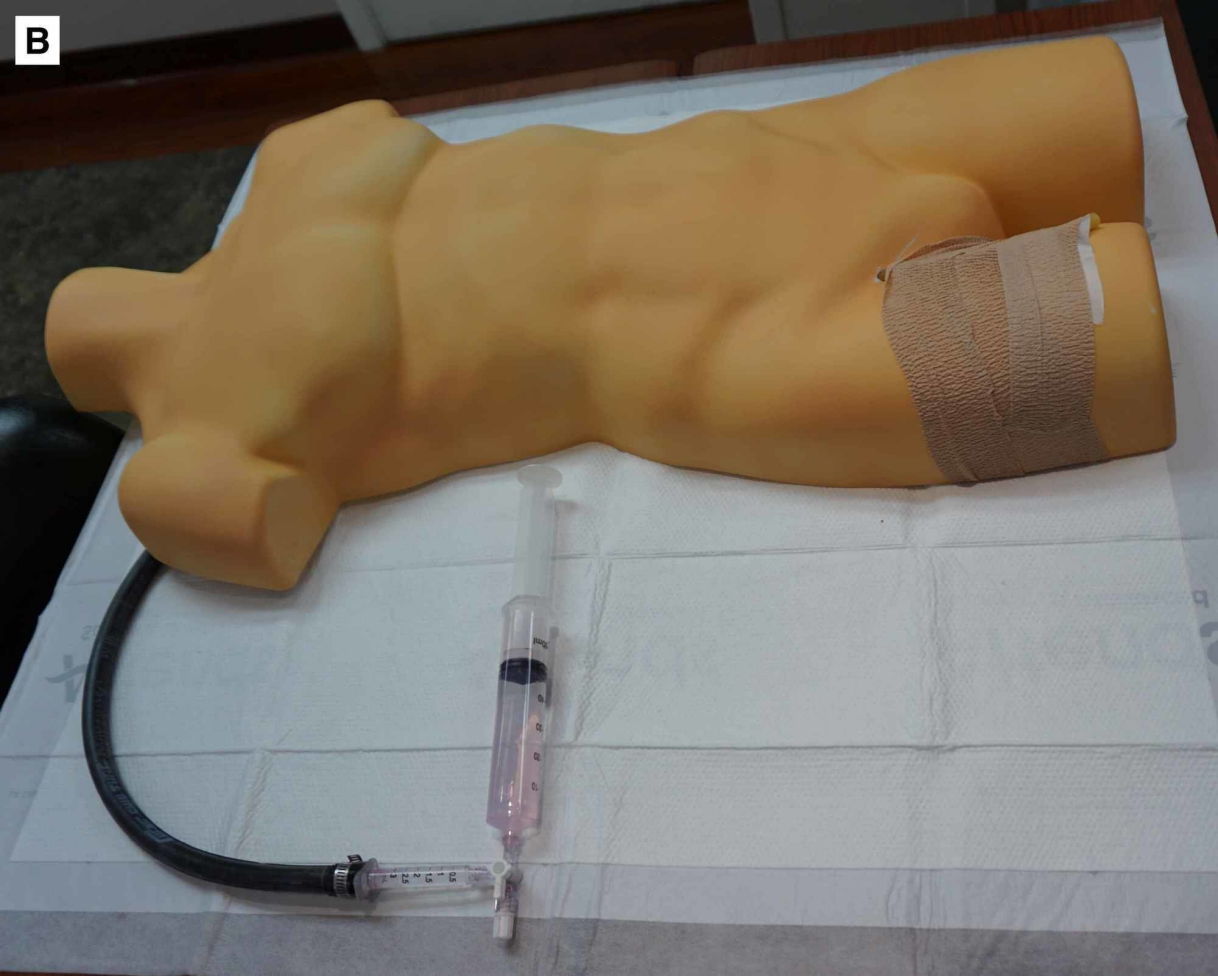
Completed REBOA insertion trainer. The rubber tubing (used to simulate the femoral artery) is covered and secured to the torso using self-adherent wrap to simulate skin. REBOA, resuscitative endovascular balloon occlusion of the aorta

**Figure 5:**
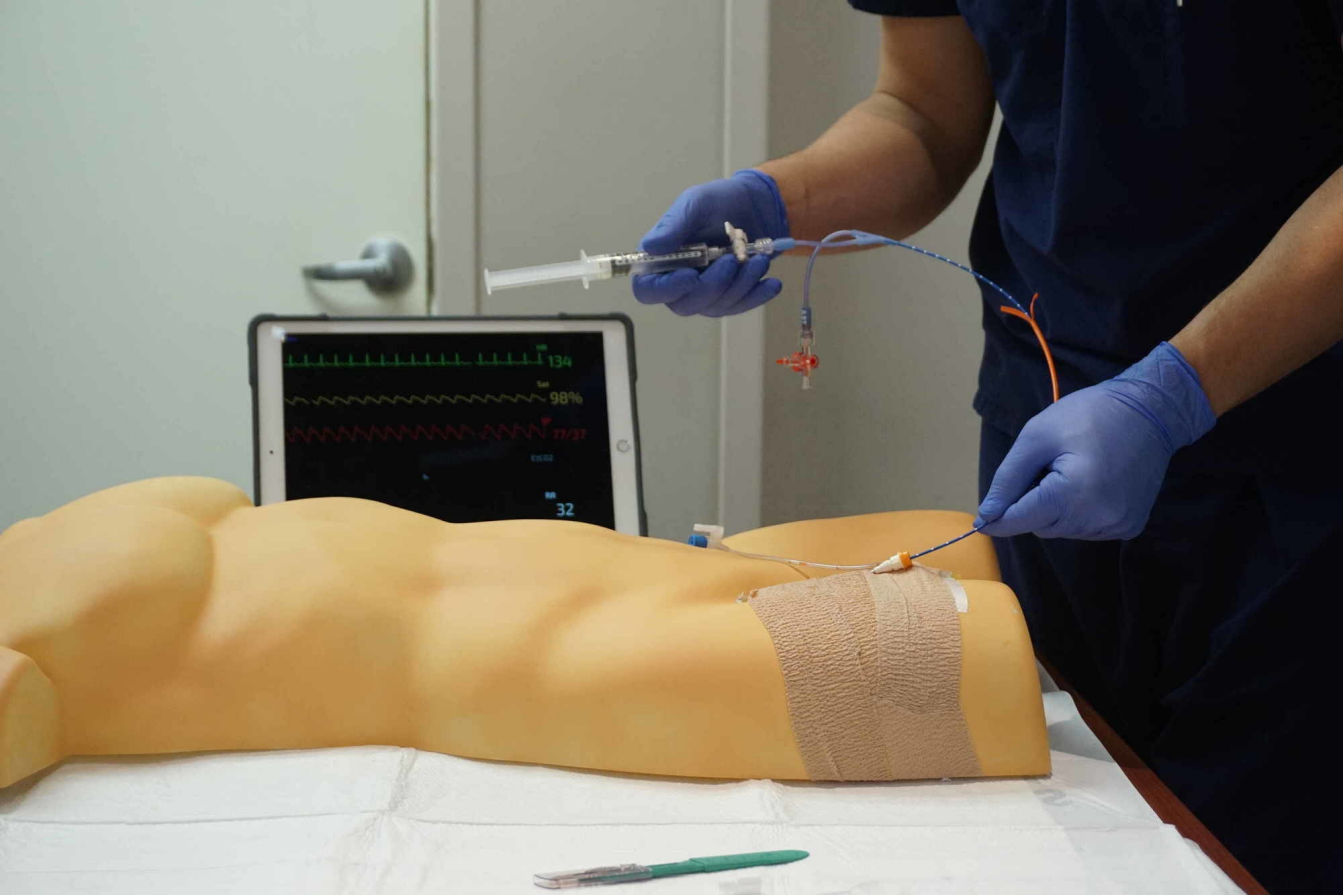
REBOA insertion trainer in use. The participant is advancing the ER-REBOA catheter through the introducer sheath in the simulated femoral artery. REBOA, resuscitative endovascular balloon occlusion of the aorta

The simulated aorta and femoral artery were then attached to a human torso mold allowing for measurement of REBOA placement depth (Figures 3, 4). The femoral artery was covered and secured to the torso with self-adherent wrap to simulate skin. This allowed for arterial puncture and placement of the introducer sheath as well as ER-REBOA catheter (Figure 5). The system was pressurized during simulation using a 60 ml syringe connected to the three-way stopcock. After needle puncture of the rubber tubing (femoral artery) was performed, a repetitive plunging action was applied to the 60 ml syringe to generate a pulsatile flow of liquid and give the appearance of an arterial stick. Because the Seldinger technique was used, there was a reasonably brisk leak from the rubber tubing (femoral artery) at the site of the introducer sheath placement once the sheath was removed. After each needle stick, a hemostat or similar clamp was used to clamp just cephalad to the leak allowing the model to be used multiple times with minimal reset time between learners. This also maintained the closed system and allowed the model to remain pressurized.

## Discussion

A total of 21 EM residents participated in the procedural session, with representation from all three residency classes. Residents completed anonymous paper surveys in which they rated aspects of the session on a five-point Likert scale and provided comments. Participants rated the fidelity of the REBOA insertion trainer very highly (mean = 4.05, SD 0.67) and felt that the training was overall very useful (mean = 4.29, SD 0.56). Comments regarding the model included, “It was great to use the actual ER-REBOA^TM^ kit! Very fun to learn this skill that we never really get to practice.” One resident wrote, “Excellent and useful - thanks!” while another wrote, “The model felt realistic. Awesome!”. There were no negative comments. 

Our model is limited by the ease of arterial stick as well as the lack of a palpable pulse likely due to the thickness of the natural rubber tubing. Given that the goal of our model was to provide training on REBOA and not specifically arterial line placement, we feel the benefit of the model significantly outweighs these minor limitations.

This is a low-cost model that can be quickly and easily assembled with readily available materials, making it ideal for military medical readiness and skill maintenance, particularly in the deployed and austere setting. Its value is not limited to low resource environments however, as it has demonstrated value in a large level 1 academic center and should also be considered for incorporation by military treatment facilities (MTFs). 

As educators, we are always looking for more realistic training models for our residents and active duty providers. Because REBOA insertion is a HALO event, many residents and staff physicians alike will rarely encounter this scenario. We have created a low cost REBOA task trainer that is easy to build, reusable, and portable, and can be utilized either in a hospital or austere training environment. Other EM residencies, MTFs, and military units are encouraged to use this model in their own programs to facilitate training of the REBOA procedure, and we hope that surgery training programs will find it useful as well.

## Conclusions

REBOA placement is a HALO event with the potential to save lives. Gaining and maintaining skills at REBOA placement, however, is challenging due to rarity of the procedure and limited training opportunities. We describe a low-cost REBOA simulation trainer that can be assembled from inexpensive and readily available materials. This simulation trainer has been well received by residents training in a level 1 trauma center, and would also be an ideal model for training in austere and resource-limited settings to include the deployed environment. 

## References

[1] Kisat M, Morrison J, Hashmi Z, Efron D, Rasmussen T, Haider A (2013). Epidemiology and outcomes of non-compressible torso hemorrhage. J Surg Res.

[2] Brenner M, Teeter W, Hoehn M (2018). Use of resuscitative endovascular balloon occlusion of the aorta for proximal aortic control in patients with severe hemorrhage and arrest. JAMA Surg.

[3] Moore LJ, Brenner M, Kozar RA (2015). Implementation of resuscitative endovascular balloon occlusion of the aorta as an alternative to resuscitative thoracotomy for noncompressible truncal hemorrhage. J Trauma Acute Care Surg.

[4] DuBose JJ, Scalea TM, Brenner M (2016). The AAST prospective Aortic Occlusion for Resuscitation in Trauma and Acute Care Surgery (AORTA) registry: data on contemporary utilization and outcomes of aortic occlusion and resuscitative balloon occlusion of the aorta (REBOA). J Trauma Acute Care Surg.

[5] Romagnoli A, Teeter W, Pasley J (2017). Time to aortic occlusion: it’s all about access. J Trauma Acute Care Surg.

[6] Brenner M, Bulger E, Perina DG (2018). Joint statement from the American College of Surgeons Committee on Trauma (ACS COT) and the American College of Emergency Physicians (ACEP) regarding the clinical use of resuscitative endovascular balloon occlusion of the aorta (REBOA). Trauma Surg Acute Care Open.

[7] Greene J (2018). Who should perform REBOA technique? Guideline limiting emergency physician use draws fire. Ann Emerg Med.

[8] Brenner M, Inaba K, Aiolfi A (2018). Resuscitative endovascular balloon occlusion of the aorta and resuscitative thoracotomy in select patients with hemorrhagic shock: early results from the American Association for the Surgery of Trauma’s Aortic Occlusion in Resuscitation for Trauma and Acute Care Surgery Registry. J Am Coll Surg.

[9] (2020). Prytime Medical Simulation Trainer for Arterial Access and REBOA. https://prytimemedical.com/product/staar/.

[10] Prytime Medical (2020). Prytime Medical. The REBOA Access Task Trainer (RATT) pulsatile simulator assemble, disassemble & troubleshooting instructions. http://prytimemedical.com/wp-content/uploads/2018/04/RT9200-01-Rev-A_RATT-Assemble-Disassemble-Troubleshooting-Instruction.pdf.

